# A Review of Refractive Errors Post Anti-Vascular Endothelial Growth Factor Injection and Laser Photocoagulation Treatment for Retinopathy of Prematurity

**DOI:** 10.3390/jcm14030810

**Published:** 2025-01-26

**Authors:** Amy T. Wang, Isha Gupta, Shuan Dai

**Affiliations:** 1 Department of Ophthalmology, Queensland Children’s Hospital, South Brisbane 4101, Australia; amytwang@alumni.ubc.ca (A.T.W.);; 2 Faculty of Medicine, The University of Queensland, St Lucia 4067, Australia

**Keywords:** retinopathy of prematurity, refractive errors, myopia, anti-vascular endothelial growth factor, laser photocoagulation

## Abstract

**Background/Objectives**: The aim of this study was to examine the incidence and severity of refractive errors that occur following the treatment of retinopathy of prematurity (ROP) with anti-vascular growth factor (anti-VEGF) agents and laser photocoagulation. **Methods**: A review of the literature using three databases (PubMed, Embase, Medline) was performed using appropriate search terms, and the results of the relevant studies were compiled and extracted for descriptive analysis. **Results**: Sixty articles were identified. The cohorts in the studies were treated with either anti-VEGF monotherapy, laser photocoagulation, or a combination, with a high prevalence of myopia, ranging from 0 to 47.7%. Refractive errors of myopia, hypermetropia, astigmatism, and anisometropia were considered in infants who received ocular interventions for ROP. **Conclusions**: In comparison to laser photocoagulation, anti-VEGF monotherapy appears to yield lower levels of myopia and anisometropia; however, the incidence of hypermetropia and astigmatism is variable among cohort groups treated with different anti-VEGF agents.

## 1. Introduction

Retinopathy of prematurity (ROP) is a proliferative retinal vascular disorder affecting premature infants with lower gestational age and birthweight. Laser photocoagulation (LPC) therapy to the avascular retina has been the gold-standard treatment for ROP as it is thought to prohibit abnormal retinal angiogenesis by laser-induced destruction of the avascular retina [1]. LPC is associated with the development of high myopia [2]. Since the BEAT-ROP (Bevacizumab Eliminates the Angiogenic Threat of Retinopathy of Prematurity) study, intravitreal anti-vascular endothelial growth factor (VEGF) injections have been used more extensively in the treatment of ROP, as they not only directly prohibit VEGF, an agent that influences retinal vascular angiogenesis and proliferation, but also facilitate normal retinal vasculogenesis [3,4]. There are several anti-VEGF agents available, and they differ in mechanism of action and dose, which results in differing systemic exposure levels that have implications on organogenesis [5,6]. Anti-VEGF is thought to be less likely to cause myopia in children treated for ROP as compared with LPC [3,5,7]. Despite this, there is conflicting information in the literature concerning the incidence and severity of refractive errors post anti-VEGF for ROP. Analysing the severity and prevalence of refractive errors in infants treated with anti-VEGF for ROP better informs clinicians of the potential risks and benefits of anti-VEGF treatment compared to laser photocoagulation or non-treatment.

The purpose of this review is to examine the incidence and severity of refractive errors that occur post anti-VEGF injections for the treatment of ROP with consideration of confounders including birthweight, length of follow-up, cohort size, ethnicity, and severity of ROP. Parameters that will be considered include spherical equivalent (SE), best-corrected visual acuity (BCVA), myopia, hypermetropia, astigmatism, and anisometropia.

## 2. Material and Methods

Three databases were used for the literature search: Embase, Medline, and PubMed. The following key search terms were utilised, limited by keyword: ‘refractive error’ OR ‘myopia’ OR ‘astigmatism’ AND ‘retinopathy of prematurity’ AND ‘anti-VEGF’ OR ‘laser’. Articles published within the past ten years were considered, and the search date was 23 June 2023 (Figure S1). The following information was extracted from each article: authors’ names, year of publication, title of study, country in which the study originated, study design, cohort size, average gestational age and birthweight of cohort, severity of ROP, dose/agent of anti-VEGF, maximum age of follow-up post treatment, and main outcomes. Studies written in languages other than English or focusing on the efficacy of anti-VEGF injections in treating ROP without focusing on refractive outcomes were excluded. 

## 3. Results

Sixty publications were identified as relevant to the topic and included in this review (Table 1). This amounted to 1408 infants and 11,283 eyes (included studies were not consistent in terms of reporting results as infants or eyes). The articles included consisted of retrospective studies (26), prospective studies (10), case series (7), systematic reviews and reviews (5), comparative studies (4), dose de-escalation studies (3), case–control studies (2), case reports (2), and an opinion piece (1). 

### 3.1. Best-Corrected Visual Acuity (BCVA)

In three studies with cohort sizes of <50 children treated with LPC versus bevacizumab or ranibizumab injections, there were no reported significant differences in BCVA after 5 years of follow-up [37,39,45]. All three studies concluded that anti-VEGF led to better other refractive outcomes than LPC [37,39,45]. Murakami et al. noted that children with zone 1 stage 1 or 2 ROP were more likely to be treated with bevacizumab than LPC [45]. Comparatively, a Japanese study examining 264 eyes found that anti-VEGF injections were associated with better BCVA and smaller SE in children with zone 1 ROP after 4 years of follow-up [46]. These findings were not reproducible in children with zone 2 ROP [46]. In contrast, another study suggested that although there was no myopic shift, there was overall worse BCVA in ranibizumab-treated type 1 ROP compared to children with type 2 ROP or no ROP after 4–6 years of follow-up [17]. Anecdotal evidence shows variation in BCVA following anti-VEGF and laser interventions. A case study examining Korean twins with A-ROP reported drastically different BCVAs after 10 years of follow-up. One of the children had received two rounds of intravitreal bevacizumab and the other child had a combination of laser with intravitreal bevacizumab [31]. The child that solely received injections had better BCVA than the laser-treated child, who also had high-grade myopia and astigmatism [31]. Another case study that examined Chinese triplets with stage 3 zone 2 ROP with plus disease had variable outcomes in refractive errors [58]. Of the triplets, two received bevacizumab and only one developed severe myopia [58]. 

### 3.2. Spherical Equivalent (SE)

A systematic review completed in 2022 evaluating two randomised control trials (RCTs) and twenty-two observational studies comparing anti-VEGF and LPC in refractive outcomes suggested that there was increased myopic SE in the LPC groups compared to the anti-VEGF groups [7,59]. Tsiropoulos et al. concluded, based on three observational studies comparing bevacizumab, ranibizumab, and LPC, that there was no difference in SE amongst the three interventions [59]. An RCT examining 232 eyes included in the systematic review concluded that there was no difference in spherical or cylinder power amongst the LPC- and anti-VEGF-treated groups [59,67].

A prospective American study that examined the rate of children under a birthweight of 1000 g who had SE progression over 3.5 years noted children treated with laser had faster SE progression than those treated with bevacizumab, leading to more myopic final SE results [54]. The final incidence of myopia across zone 1 and zone 2 ROP from LPC was 85% compared to injections at 45% [54]. An observational study examining 101 eyes compared bevacizumab and ranibizumab and showed there was no difference in SE; however, this study had variable follow-up (the bevacizumab group was followed up for 30 months, whereas the ranibizumab group was followed up for 13 months) [33]. An Indian study examining 102 infants with 1–2 years of follow-up showed that the mean SE from LPC was between −8.25 D to +5.5 D, while those with spontaneous regression of ROP had significantly less negative SE [32]. For each 1000 laser shots, there was an associated decrease in SE by 0.3D to 0.65D based on two Indian and American studies that enrolled 100 and 150 eyes, respectively, with follow-up for 1–3 years. [29,32]. Myopic SE post LPC treatment was supported by three other retrospective studies; two of these had cohort sizes <100 and one study had a cohort size of n = 1607. Although the consensus was that SE is less myopic amongst injection-treated children than in those treated with laser, all studies had variable follow-up periods (with two studies reporting findings from 12 months of follow-up and the other from 5 years) [38,39,66]. 

### 3.3. Myopia

The RAINBOW extension study, conducted in multiple sites across Europe and Asia, demonstrated that 0.2 mg ranibizumab led to a much lower incidence of severe myopia (5%), defined as −5.0 dioptres or worse, than laser treatment (20%) in approximately 153 children who were followed up over two years [40]. Two systematic reviews and multiple observational studies, the majority of these conducted in European countries, included in this review support the conclusion that anti-VEGF injections (in particular bevacizumab) is associated with lower rates and degrees of myopia compared to laser therapy [9,10,25,46,57]. All observational studies had variable follow-up periods ranging from less than 18 months to 12 years of follow-up [29,33,37,40,66]. The two systematic reviews had follow-up periods between 9 months and 5 years [35,57]. The rates of myopia reported in an Iranian study were comparable between children who received bevacizumab injections and those with ROP that spontaneously resolved after 12 months of follow-up [49].

Two large clinical trials, the RAINBOW and BEAT-ROP studies, reported 5% and 3.8% rates of high and very high myopia following ranibizumab and bevacizumab injections, respectively, compared to LPC, which was reported as 20% and 51.4% for high and very high myopia, respectively [40]. Not all studies controlled for and reported refractive errors with respect to the zone, staging of ROP, and presence of plus disease [25,26,35,37]. Children with more severe stages and posterior zones of ROP were found to have faster myopic progression than children with less severe ROP after 1.5–3 years of follow-up [12,63]. A Turkish study on 157 eyes with 1-year follow-up analysing factors affecting the rates of myopia in ROP children concluded that stage 3 ROP was significantly associated with negative SE values, indicating that a higher stage of ROP is affiliated with a higher degree of myopia [65]. One study also reported that a confounding variable was the average age of children at follow-up after being treated with laser was older (mean age 12) than that of children treated with injections (mean age 2), which may have affected the recorded incidence of myopia [53]. Numerous studies included in this review had variable follow-up periods for the interventions analysed: some studies followed up anti-VEGF refractive errors for less time than LPC treatment and vice versa [37,63]. A Japanese study analysing 178 eyes showed that a birthweight of less than 1000 g is associated with a higher frequency of high myopia [42]. 

Amongst the five systematic reviews included, including a Cochrane systematic review, four unanimously agree that LPC is associated with a higher degree and incidence of myopia compared to anti-VEGF treatment [35,57,59,68]. Only one systematic review from 2020 analysing 3701 eyes found no significant difference in myopia incidence between LPC and anti-VEGF groups [48]. 

There are five articles that refute the conclusion that LPC causes a higher incidence and degree of myopia compared to anti-VEGF. Three studies examining variable cohort sizes (n = 54–350) of American or Asian populations found no difference in myopia (not inclusive of severe myopia) incidence amongst the anti-VEGF or laser-treated groups after approximately 3 to 9 years of follow-up [24,34,36]. Another Indian study involving 134 eyes with approximately 1-year follow-up determined that there was no significant difference in the prevalence of myopia in children treated with bevacizumab versus bevacizumab and laser [47]. One American study with a small sample size (n = 43) including patients who were treated with bevacizumab and then subsequently underwent laser therapy reported 55% myopic refraction in their cohort, as compared with 41% who had hyperopic refraction after 4 years of follow-up [51].

Amongst the anti-VEGF agents used, it appears that bevacizumab may be associated with a greater myopic shift compared to other agents, based on the results of three studies with cohort sizes of 62 to 187 eyes with a follow-up of 1 to 3 years [14,15,56]. It is important to note that these studies did not control for the severity of ROP and that two of these three studies reported on Taiwanese populations. East Asian populations are generally more prone to myopia than the average population, with a prevalence rate of 69% seen in one systematic review [69]. 

A Canadian study with a cohort of 101 eyes reported marginally higher cases of high myopia (n = 22, 21.7%) associated with bevacizumab use than low myopia (n = 18, 17.8%) after 5 years of follow-up [30]. Another American multicentre phase 1 dose de-escalation study found low-dose bevacizumab yielded high myopia in 14 children (21%) out of 120 infants after 2 years of follow-up [61]. A Turkish study examining 171 eyes found 22% of infants (n = 18) developed myopia after 18 months’ follow-up [12]. Contrastingly, two studies examining European and Hispanic populations with cohort sizes of 18 to 120 infants showed that bevacizumab is associated with only mild myopia (12 months of follow-up and 5 years of follow-up), respectively [23,41]. Birthweight was not controlled in any of the above-mentioned studies. 

Another Indian study involving 134 eyes with 1-year follow-up showed 56% of cases developed low-grade myopia and 13.4% developed high-grade myopia [47]. Similar statistics were seen in another Spanish study involving 76 eyes with 3 years of follow-up where 20% were myopic post bevacizumab usage [50]. Aflibercept was shown to cause low-grade myopia in 17 eyes after 1 year of follow-up; however, this study reported on an exclusively Asian population, which may have affected the incidence of reporting [16,69]. 

Comparatively, it appears that ranibizumab is associated with low cases of myopia, with a rate of approximately 7.2% after 1 year of follow-up in a Middle Eastern population [8]. Another study using ranibizumab with a large cohort of 186 eyes had a higher incidence of myopia, at 37.5%, and high myopia, at 3.4%, after 2 years of follow-up [43]. One literature review showed a varied incidence of high myopia following anti-VEGF treatment, ranging from 0 to 35% [44]. Therefore, the choice of anti-VEGF agent to reduce the prevalence of myopia remains unclear, though most studies are on bevacizumab. 

When anti-VEGF treatment is combined with LPC, the incidence of myopia changes drastically, with 7% of cases developing high myopia and 33% having low myopia after 2 to 3 years of follow-up in a cohort of 68 eyes in an American population [27,28]. A retrospective study in an Asian population demonstrated that, at 2 years of follow-up, severe ROP patients treated with intravitreal injections alone had a lower incidence of myopia as compared with those who received combined bevacizumab and laser treatment [13]. Another observational Turkish study examining 160 eyes with 18 months to 5 years of follow-up showed that there was no significant difference in refractive error or myopia in children treated with bevacizumab or bevacizumab and laser treatment [21]. 

### 3.4. Hypermetropia

A Canadian study showed there was a 16.8% incidence of hypermetropia following bevacizumab injections in a cohort of 101 eyes after 5 years of follow-up [30]. This was also supported by two other articles reporting on European populations, with 12-month follow-ups [20,66]. One of the studies involving 86 eyes stated that compared to LPC, bevacizumab causes more incidence of low-grade hyperopia [66]. The other study involving 87 eyes reported a 2% incidence of high hyperopia following low-dose bevacizumab [20]. Holt et al. demonstrated that there was 13% incidence of lower hypermetropia and 9% incidence of high hypermetropia in an American population post combination of ranibizumab and laser treatment after 2 years of follow-up [27]. One prospective study focusing on aflibercept usage in an Egyptian population showed that across 26 eyes, on average, most cases had developed hyperopia following 1 year of follow-up [52]. Another study utilising aflibercept with a 1-year follow-up period showed 20% of hypermetropia in 17 eyes of Asian heritage [16]. Another small retrospective case series with a similar follow-up period reports that children with zone 2 ROP had a higher incidence of hypermetropia than those with zone 1 ROP in a Turkish population [11]. This is further supported by another Spanish study of 76 eyes where 77% of children developed hypermetropia after 3 years of follow-up post bevacizumab injections [50]. It appears that the incidence of hypermetropia post injection is variable amongst different study populations and different agents. 

### 3.5. Astigmatism

There are relatively fewer studies that focus on astigmatism post anti-VEGF injections, though it appears there is mostly consensus that astigmatism incidence is lower amongst children treated with injections versus laser. One systematic review reported lower rates of astigmatism in children treated with bevacizumab for type 1 ROP [57]. Another systematic review analysing 3701 eyes yielded similar findings and recorded that there was a significantly lower incidence of astigmatism in infants treated with anti-VEGF compared to laser by −0.25 D [48]. Smaller studies with 1 to 7 years of follow-up also seem to support lower incidences of astigmatism in those treated with intravitreal anti-VEGF as compared with laser across European and Asian populations [26,64]. Two small cohort studies (n < 100) in Americans populations with 10 months of follow-up and 3.5 years of follow-up did not report any significant difference in astigmatism between those treated with injection versus laser [25,54].

### 3.6. Anisometropia

One American study (n = 40) utilising a linear regression model provided evidence that children requiring subsequent laser treatment following anti-VEGF therapy are more likely to develop anisometropic amblyopia [62]. Anisometropia rates were higher in those treated with laser compared to those with ROP that did not require intervention in an Iranian study on 24 eyes treated with laser with approximately 6–7 years’ follow-up [22]. One study in an American population with a 3.5-year follow-up timeframe suggested that anisometropia rates were lower in bevacizumab-treated groups than in laser-treated groups [54]. Comparatively, another American study reported that after low-dose bevacizumab and two years of follow-up, there was a 17% incidence of anisometropia [60]. A prospective study reported an 11% incidence of anisometropia post low-dose bevacizumab after 12 months of follow-up [20]. Another Iranian study showed that compared to children who were not treated for ROP, the incidence of anisometropia was not significantly different in those who received bevacizumab after 12 months’ follow-up [49]. One systematic review completed in 2019 suggested that more research on anisometropia prevalence and severity following anti-VEGF usage for ROP is needed, as there are very limited data available [57]. 

## 4. Discussion

There are many publications comparing the prevalence and degree of myopia between the two interventions of LPC and anti-VEGF. Although major clinical trials have identified that myopia prevalence is low in eyes treated with anti-VEGF compared to LPC (<5%), there are numerous studies and systematic reviews that state that children receiving anti-VEGF therapy can still develop myopia and even high myopia. There is no consensus on the degree of myopia, with two systematic reviews stating 1.8 D of difference between the two treatment modalities and another finding over 7 D of difference, which can severely change the prognosis of retaining vision for an ROP child. Four of five systematic reviews used a single randomised controlled trial involving 211 eyes published in 2014 to draw the conclusion that myopia is more prevalent and severe in LPC-treated groups versus anti-VEGF monotherapy [7]. Currently, there are not enough good-quality RCTs to provide clarity about the difference between the two treatments.

Among the included studies, it was noted that birthweight is not well controlled, with variations in birthweight from 600 g to over 1000 g. The follow-up periods are also variable amongst the included studies, ranging from 6 months to 17 years, and there is evidence to show that myopic progression only stabilises in ROP children after 1.5 years depending on the severity of disease [63]. Thirty-five percent of the included studies reported on an Asian population, which may skew our understanding of the prevalence of myopia in ROP children, as ethnic considerations were not taken into consideration in the reporting of prevalence. Whilst we expect that infants who are treated with anti-VEGF will have higher rates and severity of myopia than those with ROP that are not treated, most of the data did not account for birthweight, ethnicity, follow-up, and severity of ROP. There was high variability in reporting the prevalence and severity of myopia in relation to the severity of ROP. Few studies included the stage of ROP and its impact on refractive errors, and only a handful reported refractive errors in reference to A-ROP and plus disease. 

## 5. Conclusions

In comparison to laser photocoagulation, anti-VEGF monotherapy appears to yield lower levels of myopia and anisometropia; however, the incidence of hypermetropia and astigmatism is variable among cohort groups treated with different anti-VEGF agents. 

Further studies should be conducted to delineate the effect of anti-VEGF in comparison to LPC on refractive errors in children with ROP, with an emphasis on controlling confounding variables. This will hopefully aid clinicians in delivering precise best practice based on high-quality evidence. 

## Figures and Tables

**Table 1:** Study characteristics.

Author/s	Title	Site Origin	Study Design	Cohort Size	Severity of ROP	Dose/Agent of Anti-VEGF/Other Intervention	Age at Follow-Up	Main Effect
Al-Balushi et al., 2019 [8]	Ocular effects of intravitreal anti-vascular endothelial growth factor (ranibizumab) for retinopathy of prematurity attending tertiary hospital in Oman: Cross-sectional study	Oman	Retrospective cross-sectional study	166 eyes	Zone 1: 24.1%, Zone 2: 73.5%, Zone 3: 2.4%. Stage 2: 7.8%; Stage 3: 82.5%; Stage 4 AROP: 1.2%; 97.6% eyes had plus disease	ranibizumab	~12 months	6% myopia at 6 months; 7.2% myopia at 12 months
Arfat et al., 2018 [9]	Comparison of complications of intravitreal bevacizumab with laser photocoagulation for the treatment of retinopathy of pre-maturity	Ireland	Comparative study	70 infants: 35 IVB vs. 35 laser	-	bevacizumab	~6 months	IVB: 5.7% high myopia + 2.85% very high myopia Laser: 17.1% high myopia IVB + 37.14% very high myopia
Asano et al., 2023 [2]	Investigating the factors affecting myopia in retinopathy of prematurity after laser treatment	Japan	Retrospective study	33 eyes	Type 1 ROP or prethreshold ROP	diode laser vs. non-ROP eyes	~3 to 10 years	SE was significantly more myopic in the ROP group (*p* < 0.001)
Barnett et al., 2021 [10]	Complications of retinopathy of prematurity treatment	-	Opinion piece	-	ROP in Zone 1	-	-	3.8% myopia post IVB vs. 51.4% laser
Bayramoglu et al., 2022 [11]	Factors associated with refractive outcome in children treated with bevacizumab for retinopathy of prematurity: the importance of retinal vascularization	Turkey	Retrospective case series	181 infants	Type 1 ROP	bevacizumab	~22.9 months	5.1% high myopia 15.1% low myopia
Bayramoglu et al., 2022 [12]	Relationship between refractive outcomes and quantitative retinal vascularization and severity of plus disease in eyes treated with intravitreal bevacizumab	Turkey	Retrospective observational study	171 eyes	Type 1 ROP and AROP	bevacizumab	~18 months	22.2% developed myopia after IVB. Retinal zone was correlated to development of myopia
Chen et al., 2014 [13]	Refractive errors after the use of bevacizumab for the treatment of retinopathy of prematurity: 2-year outcomes	Taiwan (China)	Retrospective bicentre study	64 eyes	-	bevacizumab	~2 years	In IVB, only myopia was 47.5% and high myopia was 10%. When also treated with laser, myopia was 82.4% and high myopia was 29.4%
Chen et al., 2015 [14]	Intravitreal anti-vascular endothelial growth factor treatment for retinopathy of prematurity: comparison between Ranibizumab and Bevacizumab	Taiwan (China)	Retrospective case series	72 eyes	Type 1 ROP	ranibizumab and bevacizumab	~1 year	Higher chance of high myopia following bevacizumab
Chen et al., 2018 [15]	Refractive and Biometric Outcomes in Patients with Retinopathy of Prematurity Treated with Intravitreal Injection of Ranibizumab as Compared with Bevacizumab: A Clinical Study of Correction at Three Years of Age	Taiwan (China)	Retrospective case series	62 eyes	Type 1 ROP	bevacizumab, ranibizumab	~3 years	Higher incidence of high myopia in IVB
Chen et al., 2020 [16]	Anatomical and functional results of intravitreal aflibercept monotherapy for type 1 retinopathy of prematurity: one-year outcomes	Taiwan (China)	Prospective cohort study	17 eyes	Type 1 ROP	aflibercept	~1 year	20% had hyperopia, non-significant finding; 73.3% low myopia, 6.7% had high myopia; 12.5% anisometropia
Cheng et al., 2023 [17]	Refractive status and retinal morphology in children with a history of intravitreal ranibizumab for retinopathy of prematurity	China	Prospective study	204 infants	Type 1 ROP	ranibizumab	~4–6 years	Nil myopic shift. Poorer BCVA compared to type 2 ROP children or nil ROP
Chou et al., 2022 [18]	Refractive status, biometric components, and functional outcomes of patients with threshold retinopathy of prematurity: systemic review and a 17-year longitudinal study	Taiwan (China)	Retrospective longitudinal study	28 eyes	Threshold ROP	laser (810 nm diode laser)	~17 years	All patients with ROP had myopia (average spherical equivalent of −6.35 D, ranges from −1.25 to −12.38 D), and 12 eyes (43%) were highly myopic (spherical equivalent < −6.0 D). Threshold ROP eyes exhibited significantly poorer visual acuity (*p* < 0.001), greater cylinder refractive error (*p* < 0.001), higher corneal astigmatism (*p* < 0.001), and flatter horizontal corneal curvature (*p* = 0.01) compared with age-matched controls
Chow et al., 2022 [19]	The role of anti-vascular endothelial growth factor in treatment of retinopathy of prematurity-a current review	-	Review	-	-	anti-VEGF	-	Low chance of high myopia compared to laser
Crouch et al., 2020 [20]	Secondary 12-Month Ocular Outcomes of a Phase 1 Dosing Study of Bevacizumab for Retinopathy of Prematurity	USA	Prospective cohort study	46 eyes	Type 1 ROP	0.625 mg–0.031 mg bevacizumab	~12 months	14% high myopia 2% high hyperopia 11% anisometropia
Demir et al., 2021 [21]	Evaluation of the effect of different treatment management on refractive outcomes in severe retinopathy of prematurity	Turkey	Retrospective, non-randomised, cross-sectional, observational clinical study	160 eyes: 38 IVB + 24 laser + 16 both + 44 spontaneous regression + 38 normal	Type 1 ROP or APROP	IVB or laser or both	~18 months to 5 years	Although the mean spherical power and SE in the IVB group were lower than in the LPC group (*p* = 0.019 and 0.013, respectively), there was no significant difference between the IVB group and the IVB + LPC group (*p* = 0.541 and 0.804, respectively). In terms of mean cylindrical power and prevalence of myopia and anisometropia, there was no significant difference between the treatment groups (*p* > 0.05)
Farvardin et al., 2022 [22]	Long-term Visual and Refractive Outcomes of Argon Laser-treated Retinopathy of Prematurity	Iran	Case–control study	24 laser-treated eyes + 186 spontaneously regressed ROP eyes + 74 premature eyes of non-ROP + 286 normal eyes	Type 1 ROP	laser	~6–7 years	Anisometropia (≥1.5 diopters) was diagnosed with a higher rate in the treated cases (*p* = 0.03)
Freedman et al., 2022 [23]	Low- and Very Low-Dose Bevacizumab for Retinopathy of Prematurity: Reactivations, Additional Treatments, and 12-Month Outcomes	USA	Masked multicentre dose de-escalation study	113 eyes	Type 1 ROP	2 x low dose (0.25–0.031 mg) OR very low dose (0.016–0.002 mg) bevacizumab	12 months	Median myopia was mild (−0.31 D)
Gundlach et al., 2022 [24]	Real-World Visual Outcomes of Laser and Anti-VEGF Treatments for Retinopathy of Prematurity	USA	Retrospective interventional case series	350 eyes	Type 1 and type 2 ROP	0.625 mg bevacizumab or 0.25 mg ranibizumab	~9 years	No difference in myopia between anti-VEGF and laser
Harder et al., 2012 [25]	Early refractive outcome after intravitreous bevacizumab for retinopathy of prematurity	Germany	Prospective study	12 eyes IVB and 20 eyes laser	Zone 1 or zone 2 ROP	0.375 mg bevacizumab	~10 months	Less myopia in IVB compared to laser. Nil difference in astigmatism
Harder et al., 2013 [26]	Intravitreal bevacizumab for retinopathy of prematurity: refractive error results	Germany	Retrospective non-randomized interventional comparative study	23 IVB eyes and 26 laser eyes	Zone 1 or zone 2 ROP	0.375 mg or 0.625 mg bevacizumab	~1 year	Less myopia in IVB (−1.04 D) compared to laser (−4.41 D) Moderate myopia: 17% in IVB vs. 54% laser High myopia: 9% IVB vs. 42% laser Astigmatism was significantly lower in IVB
Holt et al., 2022 [27]	Outcomes of intravitreal ranibizumab followed by laser photocoagulation for type 1 retinopathy of prematurity	USA	Retrospective case series	70 eyes	Type 1 ROP	ranibizumab and laser	~2 years	7% high myopia, 33% low myopia, 13% low hyperopia, and 9% high hyperopia
Hoppe et al., 2022 [28]	Structural and refractive outcomes of intravitreal ranibizumab followed by laser photocoagulation for type 1 retinopathy of prematurity	USA	Retrospective observational study	68 eyes	Type 1 ROP	0.25 mg ranibizumab and delayed laser	~2.7 years	SE: −0.13 D and high myopia (7%)
Hwang. et al., 2022 [29]	Association between myopia progression and quantity of laser treatment for retinopathy of prematurity	USA	Retrospective study	153 eyes	Treatable ROP	laser photocoagulation 810 nm indirect laser with a 28 D lens to apply near-confluent spots	~37 ± 29 months	Eyes that received more laser spots had significantly greater change in refractive error over time (0.30 D more myopia per year per 1000 spots)
Isaac et al., 2022 [30]	Long-term outcomes of type 1 retinopathy of prematurity following monotherapy with bevacizumab: a Canadian experience	Canada	Retrospective observational study	101 eyes	24% zone 1 ROP + 77% zone 2 ROP	0.625 mg bevacizumab	~5 years	Myopia incidence 17.8% High myopia 9.9% Very high myopia 12.9% Hyperopia 16.8%
Jeon et al., 2021 [31]	Ten-year outcomes after initial management with laser photocoagulation versus intravitreal bevacizumab injection in a pair of identical twins with aggressive posterior retinopathy of prematurity	South Korea	Case report	2 eyes	AP-ROP	2 × 0.313 mg bevacizumab	~10 years	BCVA: 20/20 and 20/50 in IVB BCVA: 20/50 and counting fingers in laser + IVB Severe myopia + astigmatism in laser + IVB
Jossy et al., 2022 [32]	Refractive outcomes following yttrium aluminum garnet laser (532 nm green laser) in severe retinopathy of prematurity	India	Cross-sectional comparative study	51 infants in each group	Treatable ROP as per ETROP	frequency-doubled neodymium-doped yttrium aluminium garnet (Nd-YAG) laser 532 nm (green laser) vs. spontaneous regression	~1–2 years	Spherical equivalent (SE) ranged from −8.25 D to +5.50 D in Group 1 and −1.00 D to +4.00 D in Group 2. Group 1 had an incidence of 23.5% of myopia and 33.4% of astigmatism, which was significantly more than Group 2. The linear regression model predicted a decrease in SE of 0.658 D if the number of laser spots increased by 1000
Kang et al., 2018 [33]	Anti-vascular Endothelial Growth Factor Treatment of Retinopathy of Prematurity: Efficacy, Safety, and Anatomical Outcomes	South Korea	Retrospective comparative study	153 eyes	-	bevacizumab, ranibizumab	~30 months	No difference in SE
Khan et al., 2022 [34]	Refractive outcomes of patients treated for retinopathy of prematurity	USA	Retrospective study	133 infants	-	bevacizumab	~3–4 years	No difference in myopia incidence between IVB, laser or both
Kong et al., 2021 [35]	Refractive outcomes after intravitreal injection of antivascular endothelial growth factor versus laser photocoagulation for retinopathy of prematurity: a meta-analysis	-	Systematic review and meta-analysis	1850 eyes: 914 eyes with anti-VEGF and 936 eyes with laser	-	anti-VEGF vs. LPC	9 months to 5 years	Less incidence of myopia in anti-vegf treatment than laser
Kuo et al., 2015 [36]	Refractive Error in Patients with Retinopathy of Prematurity after Laser Photocoagulation or Bevacizumab Monotherapy	Taiwan (China)	Retrospective observational study	54 eyes, of which 15 had IVB	Type 1 and type 2 ROP	bevacizumab	~3 years	SE: −1.71 ± 1.27 D laser vs −1.53 ± 2.20 D in IVB. No difference in myopia cases between IVB and laser
Lee et al., 2018 [37]	Macular Structures, Optical Components, and Visual Acuity in Preschool Children after Intravitreal Bevacizumab or Laser Treatment	Taiwan (China)	Comparative interventional case series	80 eyes	Type 1 ROP	0.625 mg bevacizumab	~4.9 years	Less myopia in IVB- than laser-treated children. Same BCVA in both cohorts
Linghu et al., 2022 [38]	Comparison of intravitreal anti-VEGF agents with laser photocoagulation for retinopathy of prematurity of 1627 eyes in China	China	Retrospective non-randomised comparative study	212 eyes	Type 1 ROP or APROP: Group 1: A-ROP or zone 1, Group 2: zone 2 stage 2 or stage 3 ROP with plus disease	laser (810 nm diode laser) or anti-VEGF (bevacizumab, ranibizumab, conbercept)	~1 year	Refractive data from eyes of regressed ROP patients after 1 year of anti-VEGF injection or laser therapy were significantly different. In anti-VEGF, mean astigmatism was 0.19 ± 1.51 D, and the mean spherical equivalent was 1.8 ± 1.99 D. In laser, mean astigmatism was −0.37 ± 1.62. Statistical differences were found between these two groups. Spherical equivalents were significantly higher in eyes treated with laser than in eyes treated with anti-VEGF agents (*p* = 0.06, *p* < 0.001, respectively). No difference was found in the power of astigmatism (*p* = 0.201)
Lu et al., 2022 [39]	Refractive and biometrical characteristics of children with retinopathy of prematurity who received laser photocoagulation or intravitreal ranibizumab injection	China	Case–control study	27 eyes with IVB and 28 eyes laser	Zone 2 stage 3 ROP	ranibizumab	~5 years	SE: −2.43 ± 3.56 in laser vs −0.53 ± 3.12 in IVR BCVA: log MAR, 0.17 ± 0.14 in laser vs. 0.21 ± 0.18 in IVR
Marlow et al., 2021 [40]	2-year outcomes of ranibizumab versus laser therapy for the treatment of very low birthweight infants with retinopathy of prematurity (RAINBOW extension study): prospective follow-up of an open label, randomised controlled trial	USA, Austria, Belgium, Croatia, Czechia, Denmark, Egypt, Estonia, France, Germany, Greece, Hungary, India, Italy, Japan, Lithuania, Malaysia, Romania, Russia, Saudi Arabia, Slovakia, Taiwan (China), Turkey, UK	Prospective study	180 infants	-	0.2 mg ranibizumab	~2 years	High myopia: 5% IVR vs. 20% laser
Martinez-Castellanos et al., 2013 [41]	Long-term effect of antiangiogenic therapy for retinopathy of prematurity up to 5 years of follow-up	Mexico	Prospective, interventional, noncomparative case study	18 eyes	Type 1 ROP	bevacizumab	~5 years	VA: 20/25 + low myopia in 12/18 cases (−3.2 D)
Matsumura et al., 2022 [42]	Risk factors for early-onset high myopia after treatment for retinopathy of prematurity	Japan	Retrospective observational study	178 eyes	Type 1 ROP	laser: 180–380 mW for 300 ms	~3 years	Prevalence of myopia and high myopia was significantly higher in the treated group (59.7% and 17.9%, respectively) than in the untreated group (19.7% and 0%, respectively) (*p* < 0.001). The frequency of high myopia increased steeply in infants weighing less than 1000 g at birth
Meng et al., 2020 [43]	Refractive error outcomes after intravitreal ranibizumab for retinopathy of prematurity	China	Retrospective observational study	186 eyes	Type 1 ROP	0.25 mg repeated ranibizumab injections	~2 years	37.5% myopia and 3.4% high myopia
Mintz-Hittner et al., 2016 [44]	Review of effects of anti-VEGF treatment on refractive error	-	Review	466 eyes	-	bevacizumab, ranibizumab, or aflibercept	-	Mean SE: +0.75 D to −3.57 D High myopia from 0 to 35%
Murakami et al., 2021 [45]	Comparison of 5-year safety and efficacy of laser photocoagulation and intravitreal bevacizumab injection in retinopathy of prematurity	Japan	Prospective observational study	52 eyes: 28 eyes laser + 24 eyes IVB	-	bevacizumab	~5 years	Zone 1 low stage treated with IVB more than laser. No difference in BCVA and SE. More myopia in laser
Murakami et al., 2023 [46]	Comparison of long-term treatment outcomes of laser and anti-VEGF therapy in retinopathy of prematurity: a multicentre study from J-CREST group	Japan	Multicentre retrospective study	264 eyes: 187 eyes laser + 77 eyes IVI	Type 1 ROP/ A-ROP	-	~4 years	Better BCVA in IVI vs. laser. Higher SE in IVI than laser
Pawar et al., 2023 [47]	Refractive profile of children treated with intravitreal bevacizumab for retinopathy of prematurity	India	Retrospective study	134 eyes	Type 1 ROP	bevacizumab or bevacizumab + laser	~1 year	Major refractive error was myopia. Low-to-moderate myopia was seen in 75 eyes (56%), high myopia in 18 eyes (13.4%), emmetropia in 18.7%, and hypermetropia in 11.9% of eyes. In the control group, the majority of eyes (91.8%) had emmetropia. There was no statistically significant difference in refractive outcomes in children treated with additional laser therapy when compared to children who received only IVB therapy. The prevalence of low-to-moderate myopia was more than that of high myopia in participants with zone 1 and zone 2 ROP before treatment
Popovic et al., 2021 [48]	Intravitreal Anti-Vascular Endothelial Growth Factor Injection versus Laser Photocoagulation for Retinopathy of Prematurity: A Meta-Analysis of 3701 Eyes	-	Systematic review + metanalysis	3701 eyes: 1289 eyes IVI and 2412 eyes laser	-	-	-	Astigmatism was significantly lower following IVI compared to laser
Razavi et al., 2020 [49]	Refractive outcome of intravitreal bevacizumab injection in comparison to spontaneous regression of retinopathy of prematurity (ROP)	Iran	Prospective cohort study	87 infants: 38 IVB vs. 49 no tx	-	0.625 mg bevacizumab	~1 year	Similar myopia and anisometropia rates in IVB vs. non-treated patients. Non-significant
Riera et al., 2023 [50]	Functional results in children with retinopathy of prematurity treated with intravitreal bevacizumab	Spain	Retrospective study	76 eyes	High-risk type 1 ROP	0.625 mg bevacizumab	~3 years	Median spherical equivalent at last examination was +0.94 (RIQ: −0.25, 1.88). Two eyes were emmetropic. Fifteen eyes (20%) had myopia. Fifty-seven eyes (77%) had hypermetropria. Seventy-two eyes (97%) had some degree of astigmatism
Rubino et al., 2019 [51]	Aggressive posterior retinopathy of prematurity: Functional outcomes following intravitreal bevacizumab	USA	Retrospective study	43 eyes: 35 eyes received subsequent laser	AP-ROP	bevacizumab and laser	~4 years	55% were myopic + 41% hyperopic
Salman et al., 2015 [52]	Structural, visual and refractive outcomes of intravitreal aflibercept injection in high-risk prethreshold type 1 retinopathy of prematurity	Egypt	Prospective non-randomized interventional case series study	26 eyes	High-risk pre-threshold type 1 ROP	1 mg aflibercept	~1 year	On average, mild hyperopia at 0.75 D (range: −9.5 to +4)
Hawn et al., 2020 [53]	Long term visual and neurodevelopmental outcomes in ROP patients treated with laser photocoagulation versus intravitreal anti-VEGF therapy	USA	Retrospective observational study	24 infants: 14 laser + 10 IVB/R	-	bevacizumab or ranibizumab	2–12 years	Laser treatment led to more myopia (−8 D) than IVB/R treatment (−2 D)
Simmons et al., 2021 [54]	Longitudinal Development of Refractive Error in Children Treated With Intravitreal Bevacizumab or Laser for Retinopathy of Prematurity	USA	Prospective cohort study	IVB: 22 infants + laser: 26 infants	Type 1 ROP: stage 3+ or posterior ROP in zone 1 or zone 2	0.625 mg bevacizumab	~3.5 years	Myopia: 82.7% laser vs. 47.7% IVB SE: −8.0 D ± 5.8 D laser vs. −2.3 D ± 4.2 D IVB Rate of SE change: −5 D/year in laser vs. −3.5 D/year in IVB Low anisometropia in IVB vs. laser VA same in both cohorts
Spiller et al., 2022 [55]	Refractive outcomes after primary bevacizumab followed by laser versus primary laser alone for Retinopathy of Prematurity.	USA	Retrospective cohort analysis	28 eyes had IVB and laser vs. 297 eyes had laser only	Type 1 ROP	bevacizumab and laser	~19 months	Similar incidence of myopia and high myopia in laser +IVB vs. laser alone
Suren et al., 2022 [56]	Comparison of bevacizumab, ranibizumab and aflibercept in retinopathy of prematurity treatment.	Turkey	Retrospective study	187 eyes: 53 eyes B, 77 eyes R, 56 eyes A	-	bevacizumab, ranibizumab, aflibercept	~3 years	Myopic shift for IVB
Tan et al., 2019 [57]	Development of refractive error in children treated for retinopathy of prematurity with anti-vascular endothelial growth factor (anti-VEGF) agents: A meta-analysis and systematic review.	-	Systematic review and meta-analysis	272 eyes IVB and 247 eyes laser	Type 1 ROP	bevacizumab	-	Low prevalence of myopia, high myopia, and astigmatism in IVB treatment vs. laser
Tseng et al., 2012 [58]	Different refractive errors in triplets with retinopathy of prematurity treated with bevacizumab.	Taiwan (China)	Case report	3 infants	Stage 2–3 ROP	bevacizumab	-	One child developed myopia; the other did not
Tsiropoulos et al., 2023 [59]	Comparison of adverse events between intravitreal anti-VEGF and laser photocoagulation for treatment-requiring retinopathy of prematurity: a systematic review.	-	Systematic review	-	Treatment requiring ROP	bevacizumab, ranibizumab, aflibercept, pegaptanib, conbercept	-	More myopia in laser vs. IVI
Wallace et al., 2022 [60]	Two-year ocular and developmental outcomes of a phase 1 dosing study of bevacizumab for retinopathy of prematurity.	USA	Multicentre, dose de-escalation study	134 eyes	Type 1 ROP	0.002–0.25 mg bevacizumab	~2 years	16% high myopia + 17% anisometropia
Wallace et al., 2023 [61]	Ocular and developmental outcomes of a dosing study of bevacizumab for retinopathy of prematurity.	USA	Multicentre, phase 1 dose de-escalation study	120 infants	Type 1 ROP	0.002–0.25 mg bevacizumab	~2 years	21% high myopia
Wiecek et al., 2022 [62]	Development of Anisometropic Amblyopia in Children treated for Type I Retinopathy of Prematurity.	USA	Retrospective study	40 infants IVB vs. 48 infants laser	-	bevacizumab	~3.2 years	High incidence of anisometropia post IVB + laser
Wiecek et al., 2022 [63]	Longitudinal Change of Refractive Error in Retinopathy of Prematurity Treated With Intravitreal Bevacizumab or Laser Photocoagulation	USA	Retrospective observational study	88 infants: 40 eyes IVB, 90 eyes laser, 36 eyes both	13.% had zone 1 ROP + 36.3% had zone 2 ROP	0.5 mg bevacizumab	~3 years	No myopic progression
Wu et al., 2023 [64]	Corneal topography in preterm children aged 2 years to 12 years with or without retinopathy of prematurity	Taiwan (China)	Prospective longitudinal cohort study	131 infants	Type 1 ROP	-	~7 years	Higher incidence of astigmatism in laser treatment vs. anti-VEGF
Yenice et al., 2023 [65]	Development of myopia in laser-treated ROP infants: prematurity or laser photocoagulation?	Turkey	Retrospective study	157 eyes	Type 1 ROP	laser (810 nm transpupillary diode laser)	~1 year	No significant association was found between GA, BW, and ROP zone and SE value, while the number of laser spots (ß = −0.27 ± 0.00 D, *p* = 0.00) and stage 3 ROP (ß = −0.29 ± 0.37 D, *p* = 0.00) were significantly associated with SE value. In multivariable linear regression analysis, significant associations were found between the number of laser spots, stage 3 ROP, and SE value (ß = −0.25 ± 0.00 D, *p* = 0.01 for number of laser spots, ß = −0.28 ± 0.36 D, *p* = 0.00 for stage 3 ROP)
Yenice et al., 2022 [66]	One-year refractive outcomes after intravitreal bevacizumab versus laser photocoagulation for retinopathy of prematurity	Turkey	Retrospective cohort study	86 eyes	Type 1 ROP	0.625 mg bevacizumab	~12 months	SE: 0.8 ± 1.7 D in IVB vs. − 0.5 ± 2.0 D laser. SE was lower in zone 1 ROP than in zone 2 for IVB-treated children. There was lower incidence of low + high myopia in IVB than laser. There were more cases of mild hypermetropia in IVB than laser

## Supplementary Materials

The following supporting information can be downloaded at https://www.mdpi.com/article/10.3390/jcm14030810/s1, Figure S1: Identification of studies via databases.

## Abbreviations

The following abbreviations are used in this manuscript:

ROP	Retinopathy of prematurity
VEGF	Vascular endothelial growth factor
LPC	Laser photocoagulation
BEAT	Bevacizumab Eliminates the Angiogenic Threat of Retinopathy of Prematurity
SE	Spherical equivalent
BCVA	Best corrected visual acuity
RCT	Randomised control trial
LogMAR	Logarithm of the Minimum Angle of Resolution
IVB	Intravitreal bevacizumab
IVR	Intravitreal ranibizumab
IVI	Intravitreal injection
GA	Gestational age
BW	Birthweight
B	Bevacizumab
R	Ranibizumab
A	Aflibercept
VA	Visual acuity
