# Supplementary figures and images for: A Review of Refractive Errors Post Anti-Vascular Endothelial Growth Factor Injection and Laser Photocoagulation Treatment for Retinopathy of Prematurity
Source: J Clin Med. 2025 Jan 26;14(3):810. doi: 10.3390/jcm14030810 (PMC11818255; doi:10.3390/jcm14030810)

**Figure S1.** Identification of studies via databases.

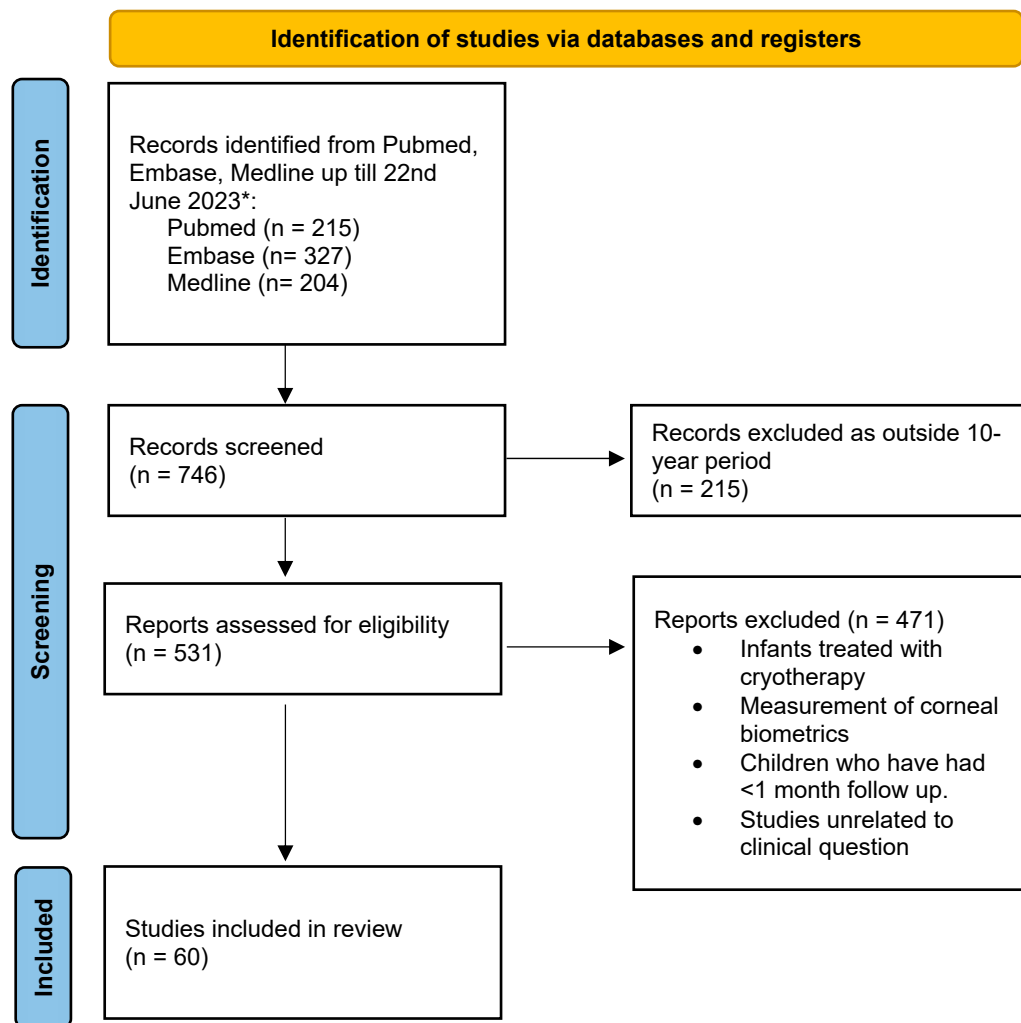

\*Search limited by keyword.

Supplement: Supplementary file 1 [file jcm-14-00810-s001.zip › jcm-3413061-supplementary.pdf]
